# Editorial: Free Fatty Acids as Signaling Molecules: Role of Free Fatty Acid Receptors and CD36

**DOI:** 10.3389/fphys.2022.862458

**Published:** 2022-03-16

**Authors:** Carlos Puebla, Eugenia Morselli, Naim Akhtar Khan, Mauricio A. Retamal

**Affiliations:** ^1^Cellular Physiology Laboratory, Instituto de Ciencias de la Salud, Universidad de O‘Higgins, Rancagua, Chile; ^2^Laboratory of Autophagy and Metabolism, Department of Physiology, Faculty of Biological Sciences, Pontificia Universidad Católica de Chile, Santiago, Chile; ^3^Physiologie de la Nutrition & Toxicologie, INSERM 1231, Université de Bourgogne, Dijon, France; ^4^Universidad del Desarrollo, Centro de Fisiología Celular e Integrativa, Clínica Alemana Facultad de Medicina, Santiago, Chile

**Keywords:** membrane physiology, G protein-coupled receptors, lipids research, cell signaling, ion channels

## Fatty Acids as Signaling Molecules

Fatty acids (FAs) come from different types of sources, for example, medium- and long-chain FAs are derived mainly from dietary triglycerides and, on the other hand, short-chain FAs are produced by intestinal microbial fermentation of dietary fiber (Ang and Ding, 2016). In our body, FAs are not only important energy sources but also, they can act as signaling molecules in several important physiological mechanisms. Interestingly, FAs are associated with the activation of two types of plasma membrane receptors. The first is a specific family of G protein-coupled receptors (GPRs), whose members were renamed as *Free Fatty Acid Receptors* (FFARs) because different FAs have been identified as their ligands. Apparently, the FFARs family shows specific affinities for different FAs according to their chain length, then medium- and long-chain FAs (6–12 and 13–21 carbon, respectively) activate FFAR1 (GPR40) and/or FFAR4 (GPR120), whereas short-chain FAs (<6 carbon) activate FFAR2 (GPR43) and/or FFAR3 (GPR41) (Kimura et al., 2020). The second type of receptor involved in FAs signaling is a long-chain fatty acid receptor called CD36. Although the main function of CD36 is described in the uptake of long-chain FAs, it is also an important lipid sensor. This receptor is also known as a fatty acid translocase, i.e., FAT (Fujitani et al., 2014).

This Research Topic was designed to bring together works in two well-defined areas; cell signaling associated with the activation of receptors to (a) short-chain FAs (SCFAs) and, (b) medium- and long-chain FAs (MCFAs and LCFAs, respectively).

## SCFAs Signaling

Since SCFAs are the result of gut microbial anaerobic fermentation, and their receptors (as, FFAR2) are reported in intestinal epithelial cells, it is expected to find SCFAs-induced signaling in a gastrointestinal environment. In this context, Perez-Reytor et al. reviewed the possible role of this FAs type (i.e., SCFAs) in the recovery process of the intestinal epithelial barrier after bacterial colonization and exposure to specific bacterial toxins. The authors propose that FFAR2 and FFAR3 (and perhaps GPR109a) could play an active role in enhancing the functions of tight junction proteins, a process that increases the intestinal barrier integrity. As such, these receptors would be interesting candidates in the treatment of intestinal epithelial damage induced by infectious diseases.

On the other hand, Carretta et al. ably continued the role of SCFAs and the signaling and expression of their receptors (i.e., FFAR2, FFAR3, and GPR109A). They describe the participation of these receptors in the maintenance of gastrointestinal health, with an emphasis on two different but related properties: anti-inflammatory and anti-tumorogenesis effects. In particular, the author reviews the clinical benefits of SCFAs in patients with inflammatory bowel disease and colon cancer.

## MCFAs and LCFAs Signaling

Different cell types express different FA receptors, thus, for example, FFAR1 is reported in the central nervous system, pancreatic ß-cells, and bone marrow-derived cells. Whereas, FFAR1 and FFAR4 are expressed in intestinal L-cells, and FFAR4 is also expressed in the hypothalamus, adipose tissue, and taste buds. These receptors (in addition to FFAR2 and FFAR3) have also been considered as possible therapeutic targets for different disorders and diseases. In this context, Secor et al. summarize the role of FFARs in liver health and disease, with a focus on the implication of these receptors as mediators of non-alcoholic fatty liver disease (NAFLD), non-alcoholic steatohepatitis (NASH), intestinal failure-associated liver disease (IFALD) and a variety of other liver disorders, thus proposed a FFARs as a therapeutic target in liver disease.

Furthermore, it was proposed by Hidalgo et al. that LCFAs, through activation of FFAR1 and FFAR4, could have active participation as modulators of immune cells function. Specifically, in this review, the authors discuss the cellular mechanism of activation of T-cells, macrophages, and neutrophils (cells part of the adaptative and innate immune response) by different LCFAs, such as palmitic acid (16:0), oleic acid (18:1, ω-9), linoleic acid (18:2, ω-6), eicosapentaenoic acid (EPA, 20:5, ω-3) and docosahexaenoic acid (DHA, 22:6, ω-3).

Finally, another way to approach to LCFAs signaling is through the multifunctional scavenger receptor CD36, which plays a role in the oro-gustatory detection of lipids (Besnard et al., 2016), and it is known to bind to various ligands, e.g., oxidized low-density lipoproteins and dietary FAs. In the intestine, CD36 is abundant on the brush border membrane of enterocytes mainly localized in the proximal intestine. Zhao et al. recapitulate current knowledge about intestinal CD36 activity, highlighting the detection of dietary lipids, the regulation of intestinal lipids uptake, synthesis and transport, and the regulation of intestinal hormones secretion.

**Figure 1:**
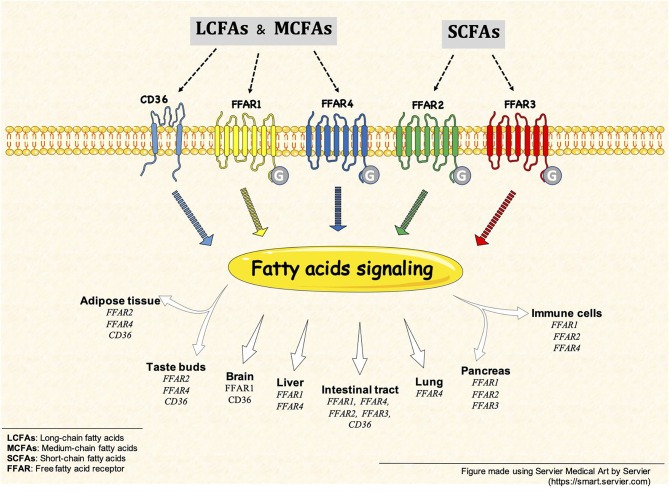
Free fatty acid receptors (FFARs) and CD36. FFARs and CD36 are activated by different fatty acid, such as SCFAs, MCFAs and LCFAs. This fatty acid signaling is present in different tissues and cell types, some example are but not limited to: intestinal tract, brain, pancreas and immune cells.

In summary, this Research Topic presents an update on the current knowledge of FFARs and CD36 receptors activity induced by FAs (i.e., SCFA, MCFAs, and LCFA) on different cell types (Figure 1), such as gastrointestinal epithelial cells, hepatocytes, and immune cells in physiological and pathological conditions. As organizers of this Research Topic, we hope to encourage the community to develop more research in this new and active field.

## Author Contributions

CP and MR wrote the original manuscript which was edited and corrected by all the authors. CP, EM, NK, and MR approved the final manuscript for publication.

## Conflict of Interest

The authors declare that the research was conducted in the absence of any commercial or financial relationships that could be construed as a potential conflict of interest.

## Publisher's Note

All claims expressed in this article are solely those of the authors and do not necessarily represent those of their affiliated organizations, or those of the publisher, the editors and the reviewers. Any product that may be evaluated in this article, or claim that may be made by its manufacturer, is not guaranteed or endorsed by the publisher.
